# Spatial proteomic mapping of the human and mouse retina using IBEX

**DOI:** 10.1172/jci.insight.204535

**Published:** 2026-04-22

**Authors:** Yuxuan Meng, Jakub Kubiak, Zuzanna Dzieniak, Lorna Fowler, Rose Avient, Jason Hopley, Linyulong Li, Chaoran Li, Yuan Tian, Bruno Charbit, Colin J. Chu

**Affiliations:** 1 UCL Institute of Ophthalmology, London, United Kingdom.; 2 NIHR Moorfields Biomedical Research Centre for Ophthalmology, London, United Kingdom.

**Keywords:** Neuroscience, Ophthalmology, Vascular biology, Molecular pathology, Neuroimaging, Proteomics

## Abstract

We generated a comparative spatial proteomic atlas of the human and mouse retina using a highly multiplexed immunohistochemistry technique called iterative bleaching extends multiplexity (IBEX). We refined the IBEX workflow by integrating an antibody dissociation option alongside chemical bleaching. This dual strategy enabled removal of the entire antibody complex, permitting the flexible use of antibodies from the same host species across iterative cycles. We coupled this workflow with super-resolution imaging via deconvolution and applied it to the retina of healthy humans and WT mice and the Crb1*^rd8^* mouse model. We successfully imaged over 25 protein markers on human and mouse tissue sections, generating spatial atlases of the major retinal cell populations. Cross-species protein expression was compared to scRNA-seq datasets to identify protein and transcript disparities. Super-resolution IBEX delineated the ultrastructural features of the outer limiting membrane (OLM), identifying CD44 as a core structural component tightly colocalized with a highly organized F-actin belt within Müller glial endfeet. Using the Crb1*^rd8^* mouse model, disruption of this complex was spatially associated with rosette formation and OLM structural failure. In summary, spatial proteomic atlases of the human and mouse retina were used to reveal insights into the arrangement of major retinal cell populations and OLM structure.

## Abstract 2

This work details a new Spatial Proteomic atlas of the mouse and human retina as a resource for scientists to study the cellular landscape of the eye in health and disease.

## Introduction

The retina is a highly organized and metabolically active neural tissue within the eye responsible for the detection of light (1, 2). Its function relies on the precise spatial arrangement and intricate interplay between its neuronal, macroglial, microglial, and vascular components (3). While single-cell RNA sequencing (scRNA-seq) revolutionized our understanding of cellular heterogeneity (4, 5), the required tissue dissociation inherently sacrifices crucial spatial information. Conversely, traditional immunohistochemistry (IHC) is typically limited to the simultaneous detection of 3–5 protein markers that fails to capture the complexity of the cellular environment and signaling networks.

To address these limitations, spatial proteomic technologies have emerged as powerful tools and named *Nature* Method of the Year in 2024. This included iterative bleaching extends multiplexity (IBEX), which has enabled the profiling of follicular lymphoma to thymic tissue (6–10). It is an established open-source technique that uses iterative cycles of antibody labeling, imaging, and chemical bleaching to visualize dozens of protein markers on a single tissue section at high resolution while preserving tissue architecture. Despite its power, the standard IBEX protocol faces challenges in flexibility. The technique relies heavily on commercially available antibodies directly conjugated to fluorophores, which can limit the availability of targets especially in underexplored tissues such as the retina. Furthermore, standard chemical bleaching (e.g., using lithium borohydride, LiBH_4_) only quenches fluorophores without removing the antibody complex. The choice of available commercial antibodies is often limited due to the host species used to generate both unconjugated and conjugated primary antibodies. Due to secondary antibody cross-reactivity, this usually prevents the use of multiple unconjugated primary antibodies from the same host species (e.g., rabbit or mouse) over different imaging cycles, severely restricting panel design.

This study addressed this challenge by establishing and validating an enhanced IBEX protocol to generate spatial proteomic maps of the human and mouse retina to enable wider discovery research and a deeper characterization of the retina. Our strategy integrated an antibody removal reagent as an alternative to chemical bleaching. This modification allowed for flexible use of the vast library of well-validated unconjugated retinal antibodies, dramatically expanding the capacity for multiplexing. We utilized this optimized platform to generate spatial proteomic maps of the healthy retina and compared them with existing transcriptomic data. Finally, we integrated super-resolution microscopy with IBEX to investigate the pathophysiology of the outer limiting membrane (OLM) in healthy humans, C57BL/6J wild-type (WT) mice, and the Crb1*^rd8^* mouse model of inherited retinal degeneration.

## Results

### A refined IBEX workflow enables generation of a spatial proteomic atlas of the healthy human retina.

**Figure 1:**
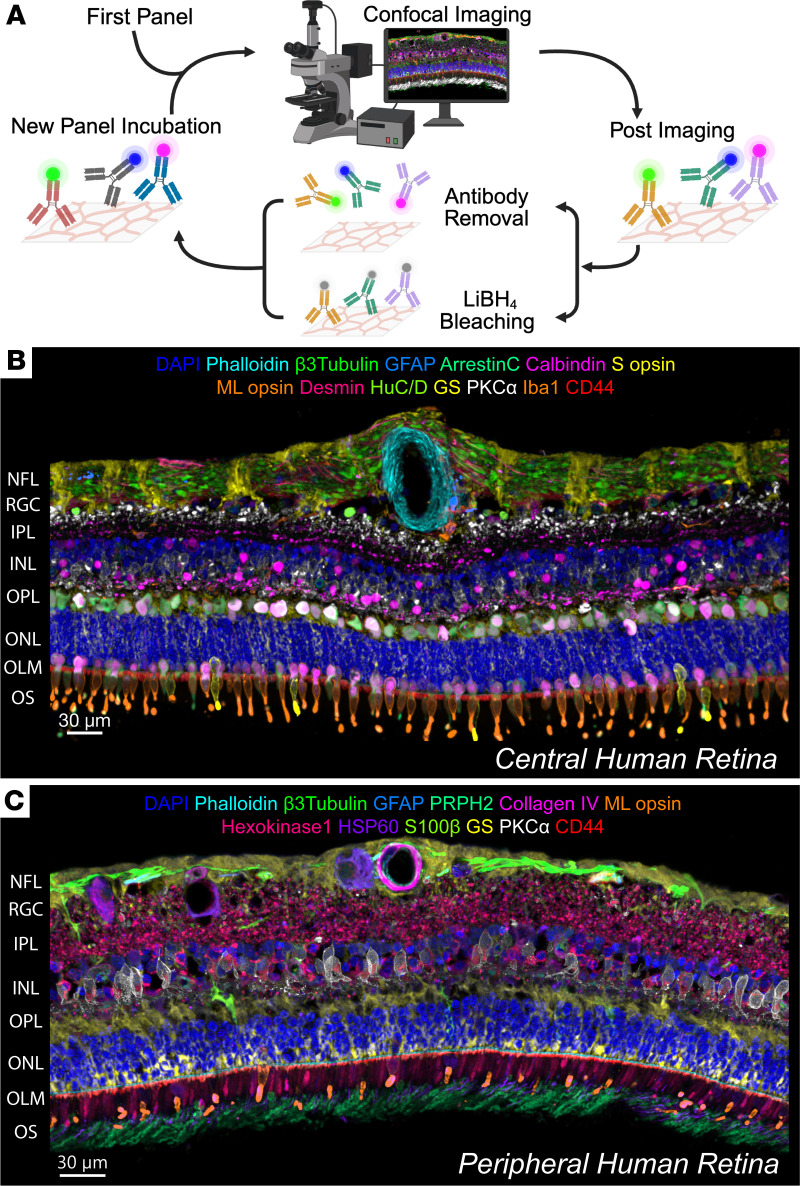
A refined IBEX protocol facilitates generation of a spatial proteomic atlas of the healthy human retina. (**A**) Schematic of the refined IBEX protocol with the inclusion of an antibody complex–removal option. (**B**) Confocal images of healthy central human retina, as visualized using the refined IBEX protocol (13 out of 23 parameters displayed). (**C**) Confocal images of a healthy peripheral human retina (13 out of 29 parameters displayed). The representative composites of the human retina show nuclei counterstained with DAPI and markers visualizing the retinal cell types spanning neuronal, glial, microglial, and vascular compartments. NFL, nerve fiber layer; RGC, retinal ganglion cell; IPL, inner plexiform layer; OPL, outer plexiform layer; OS, outer segment. Scale bars: 30 μm.

To generate a spatial protein atlas of the human retina using highly multiplexed IHC, we refined the IBEX method to build on our published work (11, 12). The existing protocol relies on chemical inactivation of fluorophores with LiBH_4_, but we now demonstrate flexible integration of a commercial antibody removal reagent into IBEX to also strip bound antibody complexes where required (Figure 1A). It overcame a core limitation of the standard IBEX protocol: the inability to use multiple unconjugated primary antibodies from the same host species (e.g., rabbit polyclonals) over different imaging cycles due to the cross-reactivity of secondary antibodies. In tissues such as the retina, for which dedicated commercial platforms or reagent panels do not readily exist, this IBEX modification facilitates greater flexibility in creating novel panels, and more accessible use of existing libraries of retina validated primary antibodies. Furthermore, expanded fluorophore options become available, as using Alexa Fluor 594, previously confirmed as resistant to LiBH_4_ bleaching, is now viable (Supplemental Figure 1; supplemental material available online with this article; https://doi.org/10.1172/jci.insight.204535DS1) (11).

Using this refined workflow, we generated an atlas of the human retina by imaging over 25 protein markers on fixed frozen sections (See Supplemental Table 1 for human-reactive antibodies), to cover the major retinal cell types in both central and peripheral regions of healthy human retina (Figure 1, B and C). We confirmed that antibody removal should be done in early panels, as after 4 consecutive rounds it can affect epitope stability.

**Figure 2:**
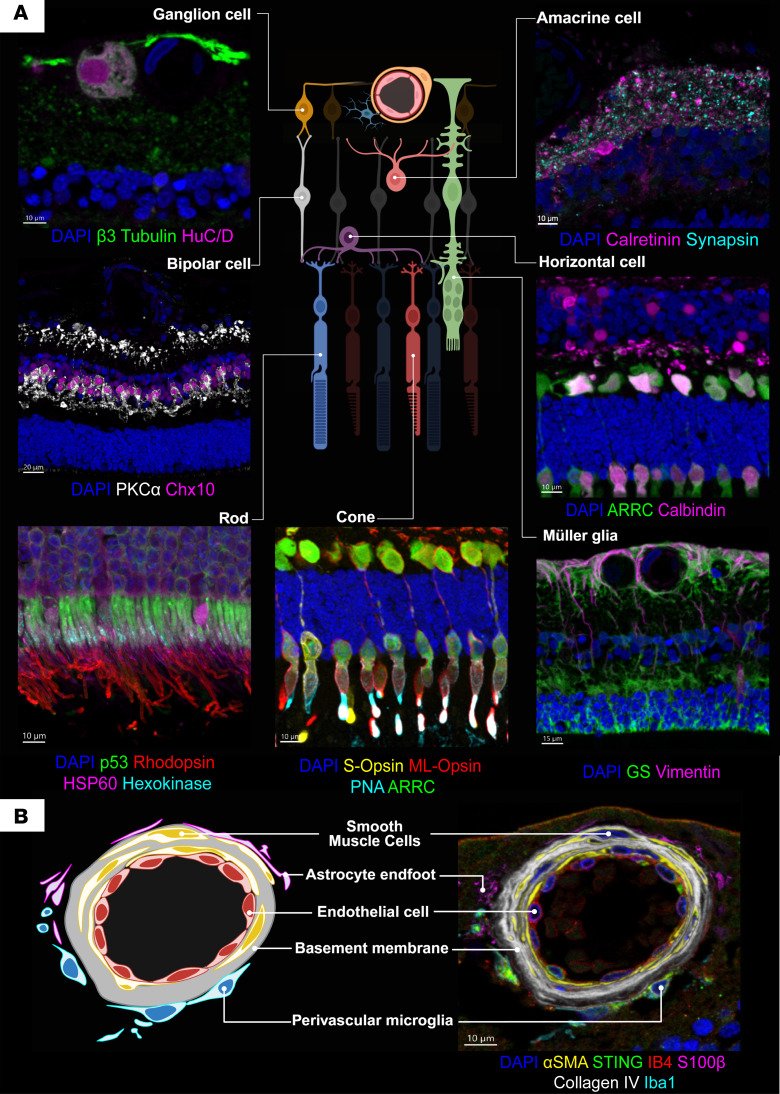
IBEX mapping of healthy human retina identifies major cell types and their respective spatial interactions. (**A**) Major cell types of the human retina displayed with relevant overlapping protein markers. All images were taken from the same tissue. Scale bars: 20 μm (middle left), 15 μm (bottom right), and 10 μm (all others). (**B**) IBEX can be used to subcategorize anatomical structures such as the perivascular unit. Images visualized from human samples first shown in Figure 1.

IBEX allowed precise simultaneous spatial visualization of the major cell types in the retina (Figure 2A) and delineated its fine laminar organization, including neuronal structures such as ganglion cells (via β3-tubulin or HuC/D), bipolar cells (PKCα or CHX10), amacrine or horizontal cells (calretinin, calbindin), and photoreceptors (ARR-C for cones and rhodopsin for rods).

Although many retinal antibody markers have been individually published, they have not been imaged in combination on the same tissue section except with 3- to 5-marker IHC, which does not allow for the simultaneous visualization of all key structural retinal cells to examine global changes and cellular interactions, which become of greater importance in heterogeneous disease states.

The neurovascular unit is a specific example of a complex retinal structure that requires multiple protein markers to be characterized, which can be achieved using IBEX (Figure 2B). To delineate the multilayered structure and interacting cells of the retinal blood vessel walls, we used isolectin B4 (IB4) to label vascular endothelial cells, collagen IV (COL IV) to outline the basement membrane, α-smooth muscle actin (α-SMA) to mark smooth muscle cells of the vessel wall, and IBA1 to identify perivascular macrophages.

### The spatial proteomic atlas of the mouse retina incorporates cross-reactive human-specific protein markers.

**Figure 3:**
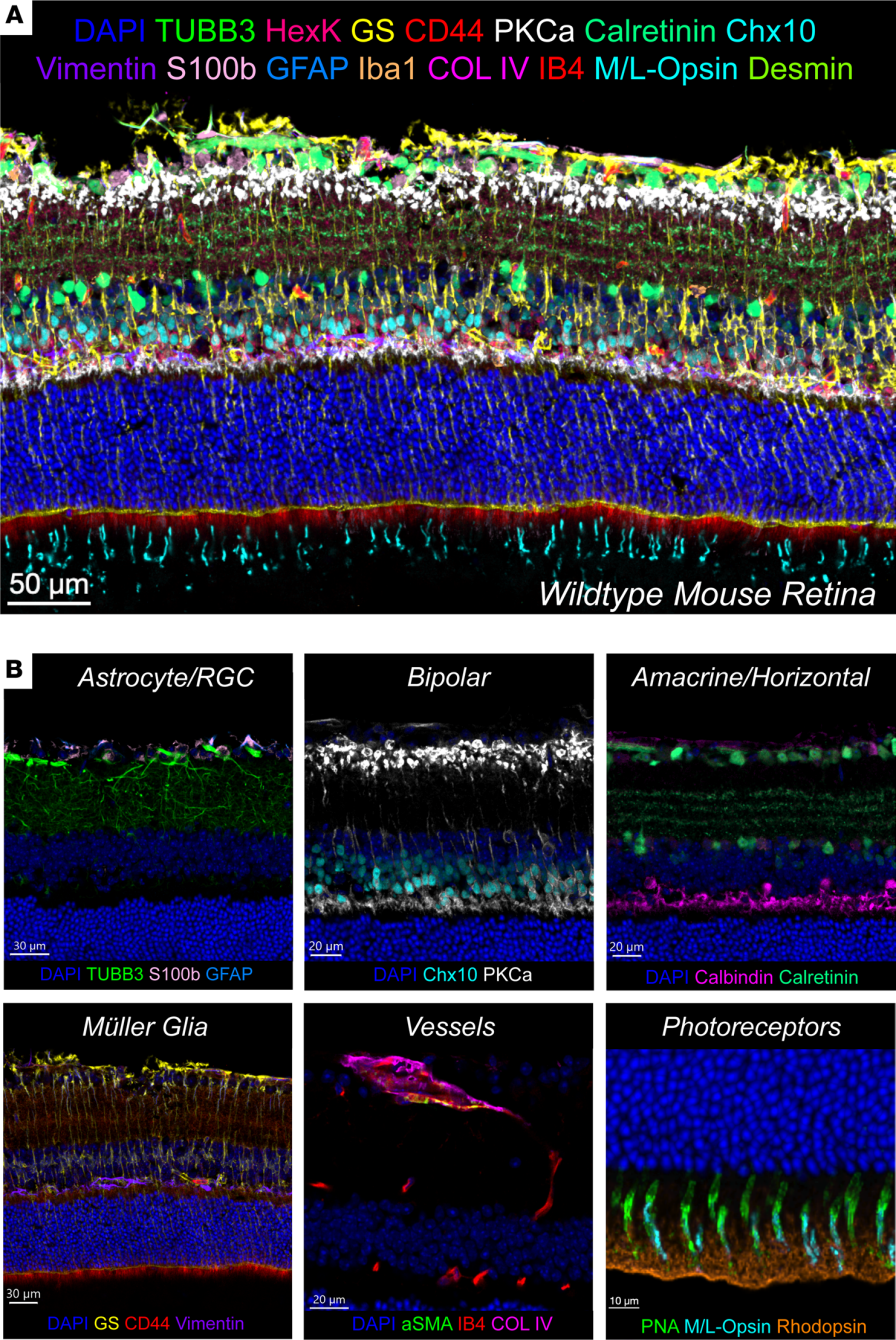
Spatial proteomic cell atlas of the mouse retina. (**A**) Confocal images of a representative healthy mouse retina generated using IBEX. Sixteen of 28 markers shown for clarity. (**B**) Magnified insets of major cell types and structures of the mouse retina displayed with relevant overlapping protein markers. Scale bars: 50 μm (**A**), 30 μm (**B**, left), 20 μm (**B**, middle and top right), and 10 μm (**B**, bottom right).

Using refined IBEX, we also generated a cellular atlas of the mouse retina, including over 20 protein markers on fixed frozen sections (see Supplemental Table 2 for mouse-reactive antibodies). Critically, we generated this atlas using 15 of the same human-reactive antibodies that exhibited similar or identical labeling patterns in the mouse (Figure 3A). Mouse retina features were consistent with the matched human retinal layers across similar physical structures and cellular subtypes (Figure 3B).

**Figure 4:**
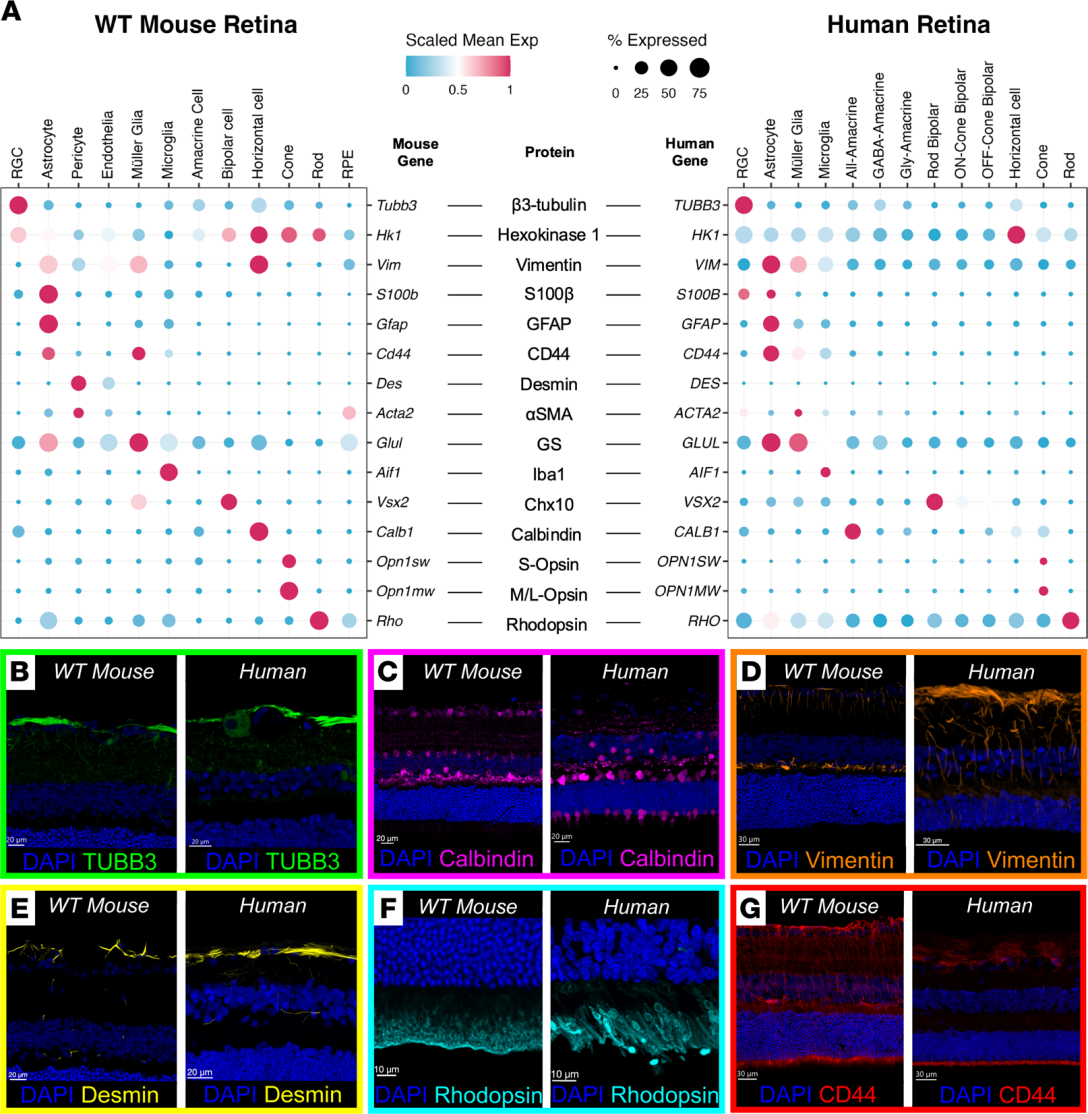
Cross-species comparison of human and mouse retina protein atlases highlights biological disparities with transcriptomic-level data. (**A**) Dot plot visualization of scRNA-seq data for mouse and human retina reconciled against cross-species-reactive antibody target proteins. (**B–D**) TUBB3, calbindin, and vimentin immunolabeling aligns with their respective transcriptomic profiles in both species. Calbindin exhibited cone labeling in humans that was not observed in mouse retina. Vimentin exhibited strong labeling at the mouse retina outer plexiform layer, corresponding to horizontal cell labeling. (**E**) Desmin protein expression was similar in both species, in contrast with that of its mRNA (*Des* in **A**). (**F**) Rhodopsin protein was uniquely detected in the outer segments only, while its RNA expression was ubiquitous. (**G**) Strong CD44 protein detection was localized at the OLM, which could not be determined using transcriptomic data. Images visualized from human and mouse samples were first shown in Figures 1 and 3, respectively. Scale bars: 20 μm (**B**, **C**, and **E**), 30 μm (**D** and **G**), and 20 μm (**F**).

To further interpret the findings, we reconciled it with published scRNA-seq datasets for both mouse and human retinas. Figure 4A compares key cross-species-reactive proteins and their corresponding transcriptional expression profiles. Mouse and human RNA transcripts and their respective proteins had broadly consistent locations of expression between species (such as IBA1, GFAP, or TUBB3; Figure 4B).

Disparities between RNA and protein across species were noted. For example, calbindin, as a canonical marker of horizontal cells, also exhibited transcriptional and protein expression within cone photoreceptors in the human retina (Supplemental Figure 2), but appeared unexpressed within the mouse. Figure 4C shows this with the human retina exhibiting calbindin immunolabeling at the outer nuclear layer (ONL) and inner segments, which is absent in the mouse.

Vimentin, classically considered a unique retinal macroglial marker, has a transcriptomic profile demonstrating high expression in horizontal cells in mice. IBEX labeling was consistent with this, revealing vimentin protein labeling at the outer plexiform layer, a feature that is absent in human tissue (Figure 4D). Colocalization within the same IBEX sample with the horizontal cell marker, calbindin, confirmed vimentin colocalization in the mouse (Supplemental Figure 3).

Desmin was not observed in any clusters within the human retina transcriptomic datasets but protein immunolabeling consistently revealed desmin localization comparable to the mouse retina, as a distinct feature around the nerve fiber layer (Figure 4E). Rhodopsin protein was only detected in the outer segments of photoreceptors (Figure 4F), in contrast with its transcriptomic profile indicating widespread rhodopsin expression in all clusters. This is a recognized technical artifact in scRNA-seq, as the structural fragility of the photoreceptors makes them sensitive to dissociation methods, potentially cross-contaminating droplets and so confounding cell clustering (13).

Finally, we observed CD44 as a marker in both mouse and human retina that was transcriptionally expressed on astrocytes and Müller glia. IBEX immunolabeling (Figure 4G) shows this to be distributed on opposing sides of the retina, with the endfeet of the Müller glial cells exhibiting concentration of CD44 ciliation near the OLM. This is a distinction that could not be elucidated from transcriptomics alone, and is a structure that is underexplored in the literature (14–18).

### Super-resolution IBEX imaging by LIGHTNING deconvolution characterizes OLM composition in human and mouse retina.

**Figure 5:**
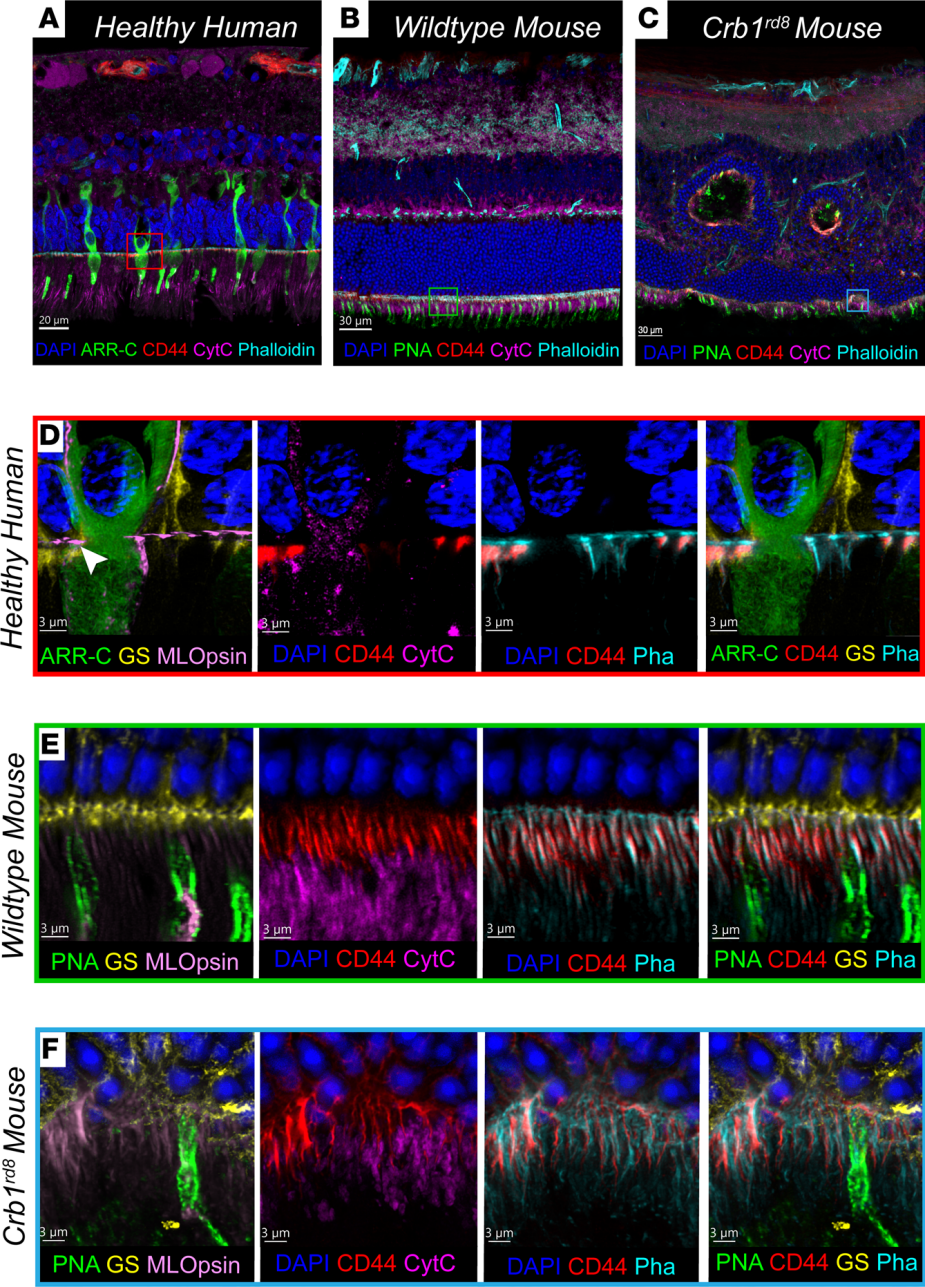
Super-resolution IBEX deconvolves the OLM retinal structure. IBEX imaging of a healthy human retina, WT mouse, and Crb1*^rd8^* mouse retina (**A–C**, respectively) visualized at standard resolution. The boxes display the areas of super-resolution imaging. (**D**) Super-resolution image of healthy human retina at the OLM region including a single cone photoreceptor. White arrow indicates the GS-phalloidin-CD44 interface. (**E**) WT mouse retina exhibited a similar OLM interface pattern, while (**F**) Crb1*^rd8^* retina, imaged underneath a rosette, has disrupted OLM structures with Müller glia retraction. Scale bars: 20 μm (**A**), 30 μm (**B** and **C**), and 3 μm (**D–F**).

We adapted the IBEX technique to integrate super-resolution confocal microscopy via Leica LIGHTNING deconvolution to characterize the composition of the OLM boundary, with a particular focus on CD44. The OLM boundary is a laminated structure defined by the adherens junctions between Müller glial and photoreceptor cells, observed in both healthy human and WT mouse retina (Figure 5, A and B, respectively). We specifically examined the Crb1*^rd8^* mouse model of inherited retinal degeneration, as it is strongly associated with OLM disruption via adhesion changes in the CRB1 complex (19).

In the Crb1*^rd8^* mouse retina, displaced photoreceptor nuclei known as rosettes are formed in the degenerative process (Figure 5C), but the dynamics of their formation and composition are incompletely characterized (20, 21). Using IBEX, we observe that rosettes are most likely invaginations occurring at the OLM and drawing photoreceptors upwards toward the inner nuclear layer — we note the presence of both rod and cone proteins within the rosettes. These rosettes also contained CD44 and phalloidin, providing evidence that this may be a complete invagination incorporating the OLM, and not just a focal folding or degeneration of the ONL.

Using super-resolution imaging, we could compare the structural configuration of the OLM boundary between human, WT mouse, and Crb1*^rd8^* mouse retina. Healthy human and mouse retinas revealed what we believe to be previously undescribed structural feature: a distinct linear “gap” within the Müller glial apical endfeet (marked by glutamine synthetase**)** that was intensely labeled by phalloidin (indicated by arrow in Figure 5D and Supplemental Figure 4). CD44 was further found to be ciliated underneath the phalloidin band and spatially above mitochondria (marked by cytochrome *c*) and cone-specific proteins (arrestin-C, M/L-opsin). Comparative visualization with the mouse retina (Figure 5E) showcased a similar GS-phalloidin-CD44 interface, albeit the distinct “gap” structure observed in the human retina was not seen in the mouse.

The Crb1*^rd8^* model at 28 weeks (Figure 5F) manifested a clear breakdown of the GS-phalloidin-CD44 interface with Müller glial endfeet retraction, suggesting indirect spatiotemporal evidence that rosettes may be formed from the OLM pulling into the ONL. With multiplexed imaging, we observed a lower density of peanut agglutinin–positive (PNA-positive) cone photoreceptors underneath the rosettes and disruption of inner segment mitochondrial processes, aligning with the literature (21).

## Discussion

Transcriptomics has proven an important resource for the scientific community to define the cellular subtypes of the retina and individual cell state profiles. While there are increasing numbers of commercial highly multiplexed IHC platforms, such as CODEX, CycIF, or imaging mass cytometry, these typically feature antibody panels optimized for common tissue types and research areas, and have not included the retina. We now describe the use of antibody removal reagents as a refinement to the flexible and open-source IBEX protocol, which was used to generate spatial proteomic atlases of the human and mouse retina and correlated cross-reactive proteins with their respective gene transcript levels acquired from previous scRNA-seq datasets. Furthermore, we integrated a deconvolution super-resolution approach with IBEX to visualize components of the OLM in health and disease.

Our refined IBEX protocol permits the use of multiple unconjugated primary antibodies from the same host species (e.g., rabbit polyclonals) over different cycles, unlocking the use of rare niche antibodies needed for specialized tissues such as the retina. We provide a resource of validated antibodies covering the major cell types of the human and mouse retina, many using identical antibody clones for both species (Supplemental Figure 4). This work is not intended as a comprehensive or static reference atlas but is intended to evolve by linking into open-science initiatives such as the IBEX Imaging Knowledge Base, where further validated antibody markers can be deposited by the research community (22).

Our spatial proteomic atlas supplements published transcriptomic datasets. There were some marked species-dependent differences within the transcriptome that can be validated with protein immunolabeling, such as the relatively elevated expression of vimentin in horizontal cells in the mouse retina. Vimentin is conventionally considered to be a canonical marker for Müller glial cells. Shaw and Weber, however, demonstrated that horizontal cell expression was different in the mouse retina (23). Our atlas confirmed this at the protein level, providing direct spatial context and cross-validation of differences between the 2 species (Supplemental Figure 5).

Desmin, for example, had little observed expression in human retinal transcriptomic datasets but was present at the protein level. Comparatively, its expression was found in pericytes and endothelial cells in the mouse retina. This difference may be due to tissue dissociation limitations for scRNA-seq (13) or the absence of active transcription of desmin after development. However, we also cannot rule out the possibility of antibody cross-reactivity with other structural proteins, which can occur in immunohistochemical assays when target transcripts are absent. Similarly, rhodopsin transcripts have also been described as problematic in rod-rich retinal samples in scRNA-seq, with reported expression in all cell types (13). This was not consistent with protein immunolabeling in both mouse and human retina, where rhodopsin was restricted to the outer segments as expected. It was previously reported that rod transcripts can confound cell clustering in scRNA-seq due to their abundance and potential cross-contamination during single-cell encapsulation (13).

We identified that the cell adhesion molecule CD44 forms “fibrillar-like” structures at the OLM, colocalizing with the F-actin–rich adherens belt (phalloidin). Identifying all the interacting components of the OLM was challenging with conventional fluorescence confocal microscopy. Therefore, we implemented IBEX in combination with super-resolution microscopy via deconvolution. We were able to observe what we believe to be a novel feature of the OLM in human retina in the form of a structural “gap” at the endfeet of the Müller glia. Although the GS-phalloidin-CD44 complex was also seen in the mouse retina, this gap was not observed. It may reflect a species-specific structural difference or could be due to the smaller size of mouse retinal structures, which may be at the limit of detection for our imaging approach.

CD44 has been closely linked to the apical processes of the Müller glia and the extracellular matrix (14, 16) and involved in the development of the retina (17), although its role in the adult human retina is not widely understood. However, CD44 was found to be significantly upregulated in inherited retinal diseases such as retinitis pigmentosa (RP) and in mouse models of RP (18, 24). Ayten et al. showed that CD44 knockout in an RP *Pde6b^–/–^* mouse model led to significantly faster photoreceptor degeneration and decreased retinal function (18). Enrichment of CD44 at the OLM and its close association with the actin cytoskeleton suggest it may play a key role in maintaining the mechanical linkage and signalling between Müller glia and photoreceptors. We directly observed the disruption of the CD44-phalloidin complex and rosette formation in the Crb1*^rd8^* mouse model as well as the presence of OLM incorporating photoreceptor outer segment components within the rosettes. This equally suggests that the origin of rosettes forms as an invagination outside the OLM engulfing outer segment components instead of forming within the ONL (21).

The study has some limitations. Dataset generation was based on postmortem tissue samples, which are static and cannot capture the dynamic changes in cellular processes, although spatial interactions can be documented. There is greater control regarding fixation in mouse tissues, but human retinas were often fixed with a postmortem delay of at least 20 hours. This delay may impact the preservation of sensitive ultrastructures, such as the junctional complexes of the OLM. Therefore, we cannot rule out the possibility that certain structural discontinuities observed in human samples, but absent in mice, may partially reflect postmortem artifacts rather than intrinsic biological differences. The number of human donor samples is always limited and heterogeneous, so larger future cohorts will be required to validate our findings. Additionally, any method based on IHC has potential issues with antibody specificity and off-target binding, particularly in human tissues, where antibodies are difficult to validate. This is another reason our study included a mouse retina atlas, in addition to being a good reference for mouse models of disease, as cross-species validation using identical clones adds cell-specific assurance. Mouse model knockouts could be used for precise protein validation to confirm their binding in human samples by proxy.

In summary, this research demonstrates the potential of spatial proteomic mapping for the study of the retina. By establishing normative datasets and validated antibody panels, the technology can be applied to other retinal diseases, such as age-related macular degeneration and diabetic retinopathy to reveal new pathological insights. Further technical developments to combine spatial proteomics with other modalities, such as spatial transcriptomics, will enable us to construct integrated multidimensional atlases of the retina in health and disease to advance biological understanding and impact ophthalmic clinical practice.

## Methods

### Sex as a biological variable.

Biological sex in both human and mouse samples was not considered as a biological variable in this study.

### Mice.

C57BL/6J mice, used as the WT control, and Crb1*^rd8^*/J mice were obtained from The Jackson Laboratory (strain nos. 000664 and 003392, respectively).

### Tissue cryopreservation and sectioning.

Human tissues were fixed with 4% paraformaldehyde for 24 hours at the NHS Blood and Transplant (NHSBT) before being exchanged into PBS. Whole eye globes, without the cornea, were shipped to the UCL Institute of Ophthalmology. Crb1*^rd8^* mice were culled after 28 weeks. Mice underwent cardiac perfusion with 2% paraformaldehyde (~10 mL per mouse) for 5 minutes before their eyes were removed. Retinas were placed in 4% paraformaldehyde after fixation before the tissue grossing. Both species of retinal tissues were fixed overnight at 4°C in a 1:4 dilution of BD Cytofix (BD Biosciences). Subsequently, tissue samples were cryoprotected by sequential incubation in 15% and 30% sucrose solutions. Tissues were embedded in O.C.T. compound (Cell Path), snap-frozen on dry ice, and stored long-term at –70°C. To ensure strong adhesion of tissue sections during the multiple washing and bleaching cycles, SuperFrost Plus microscope slides (VWR) were coated with chrome alum gelatin. Frozen tissue blocks were sectioned at 15 μm thickness using a Leica CM1950 cryostat and mounted on the coated slides.

### Optimized IBEX procedure: immunolabeling, imaging, and signal clearing.

Our iterative multiplexed IHC technique is based on the IBEX method developed by Radtke et al., with optimizations (11). The brief workflow was as follows: A standard IHC blocking protocol was used. Antibodies were diluted according to their respective validated dilutions (see Supplemental Table 1 for human, and Supplemental Table 2 for mouse) in a base of PBS plus 0.1% Tween 20 (MilliporeSigma, P9416). The antibody mix was subsequently applied to the samples, and these were left overnight in a humidified chamber at 4°C.

Most primary antibodies were acquired commercially conjugated. Some acquired purified primary antibodies were conjugated with Antibody Labeling Kits from Thermo Fisher Scientific, or secondary antibodies were still used for purified primary antibodies only. Secondaries antibodies (see Supplemental Table 3) were diluted 1:1000 in PBS and applied to the slides for 1 hour at room temperature in a darkened humidified chamber, limiting ambient light.

### Confocal microscopy.

A Leica TCS SP8 HyVolution (Leica Microsystems) confocal microscope with 40× objective was used to image mouse and human samples. Super-resolution imaging of the OLM was imaged on a Leica Stellaris 8 microscope using a 63× objective. When imaging with the 63× objective, a 6× optical zoom was applied for mouse section imaging and a 5× optical zoom for human section imaging. Leica LIGHTNING deconvolution mode was used when imaging the OLM to preserve optimal resolution and signal-to-noise ratio while imaging in high magnification.

### Bleaching and antibody removal.

Slides were placed into PBS/0.1% Tween 20 for coverslip removal. Depending on the panel design, fluorophores were either chemically inactivated or a VectaPlex Antibody Removal Kit (Vector Laboratories, VRK-1000) was used. Chemical inactivation was carried out using a 1 mg/mL solution of LiBH_4_ (Strem Chemicals, 93-0397) in deionized water. The bleaching solution was applied to the tissue twice for 10 minutes each under bright ambient room light and washed 3 times for 10 minutes each with PBS.

### Image registration and analysis.

Images from each cycle were processed using Imaris software (version 10.2, Imaris Software, Bitplane, Oxford Instruments) for preliminary processing, such as channel naming and Gaussian filtering. Subsequently, an open-source registration software based on SimpleITK was used to precisely align all imaging cycles into a single coordinate system using DAPI or Hoechst nuclear stains as fiducial markers, generating a single multichannel image file (25). Quantitative analysis (e.g., fluorescence intensity measurements) was performed using ImageJ/Fiji software or Imaris.

### scRNA-seq data.

The scRNA-seq datasets were obtained from publications for human (26) and mouse (27) retinas and visualized using the Broad Institute Single Cell Portal (https://singlecell.broadinstitute.org/single_cell). Precalculated mean expression values and the percentage of cells expressing specific genes were extracted directly from the provided source tables and reformatted using using R Statistical Software (version 4.5.1; R Core Team 2025) for visualization. No raw data reprocessing or additional bioinformatics pipeline analysis was performed.

### Statistics.

All statistical analyses were performed using Prism (version 9.0.0, GraphPad Software). For comparisons between 2 independent groups, the unpaired, 2-tailed unpaired Welch’s *t* test was used for data that passed the normality test. Data are presented as the mean ± standard error of the mean (±SEM). A *P* value of less than 0.05 was considered statistically significant.

### Human and mouse tissue acquisition and ethics approval.

Postmortem human retinal tissue for this study was obtained through Moorfields Biobank (NHS REC approval: 20/SW/0031), with informed consent from donors and eye retrieval provided by NHSBT. All animal procedures were performed under a UK Home Office project license (PP1506797) and adhered to the ARVO Statement for the Use of Animals in Ophthalmic and Vision Research.

### Data availability.

Files are accessible to download from https://doi.org/10.5281/zenodo.17975750

## Author contributions

YM, JK, and CJC conceived and designed the study. YM, JK, ZD, LF, RA, and BC performed the experiments. YM, JK, and JH conducted image processing and data analysis. ZD, JH, LL, CL, LF, RA, YT, and BC provided technical support and reagents. YM, JK, and CJC wrote the manuscript. All authors reviewed and approved the final manuscript.

## Conflict of interest

The authors have declared that no conflict of interest exists.

## Funding support

CJC is supported as a Wellcome Clinical Research Career Development Fellowship (224586/Z/21/Z), which funded this work.

- JK is supported by The Macular Society funded by The Albert Gubay Foundation (23-PhD-02).
- RA, JH, and YT were supported by Moorfields Eye Charity (GR001504, PhD-24-105, GR001475).
- This work was also funded by the National Institute for Health and Care Research (NIHR)
- Biomedical Research Centre at Moorfields Eye Hospital NHS Foundation Trust and UCL
- Institute of Ophthalmology.
- The views expressed are those of the authors and not necessarily those of the NHS, the NIHR or the Department of Health and Social Care.

## Supplementary Material

Supplemental data

## Version 1 04/22/2026

Electronic publication
