# Supplementary material for: Spatial proteomic mapping of the human and mouse retina using IBEX
Source: JCI Insight. 2026 Apr 22;11(8):e204535. doi: 10.1172/jci.insight.204535 (PMC13135386; doi:10.1172/jci.insight.204535)
Supplement: Supplemental data [file jciinsight-11-204535-s023.pdf]

Supplementary Data

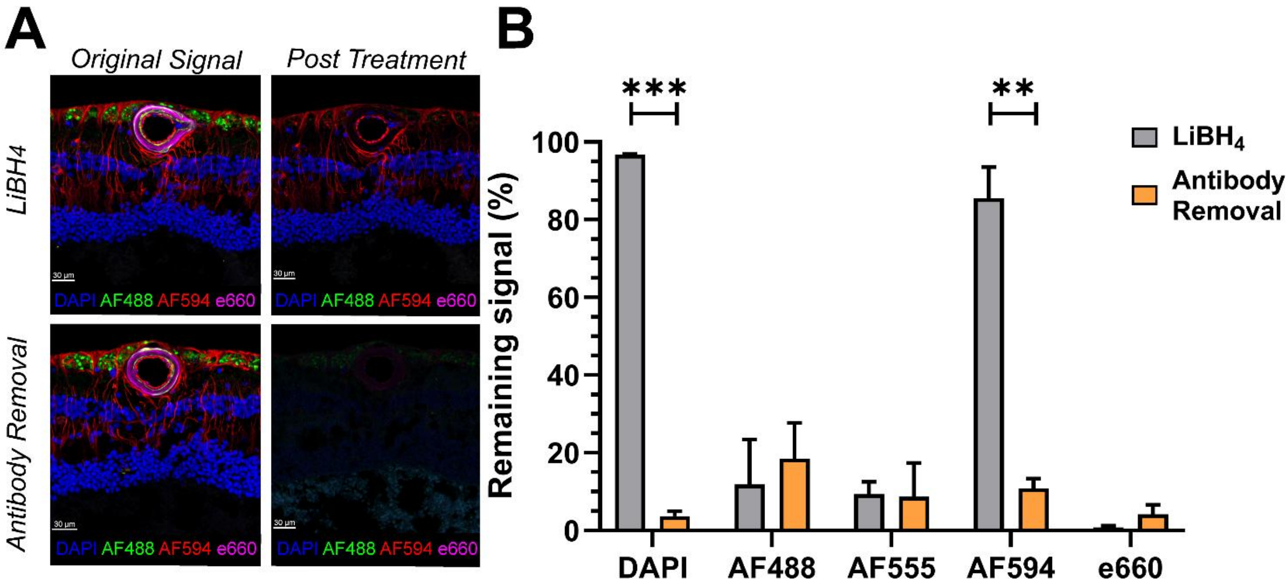

Figure S1: Antibody removal reagent significantly diminishes fluorescence signal from DAPI and AF594 which is resistant to $LiBH_4$ bleaching. (A) DAPI or AF594 is not bleached by $LiBH_4$ treatment. Antibody removal reagent attenuates their fluorescence. Scale Bars: 30µm **(B)** Percentage of fluorophore signal remaining after 20 minutes of $LiBH_4$ or antibody removal reagent treatment. Mean ± SEM, n=3 different human retinas. DAPI and AF594 signal is significantly attenuated by antibody removal compared to $LiBH_4$ (T-test: **p<0.01, ***p<0.001)

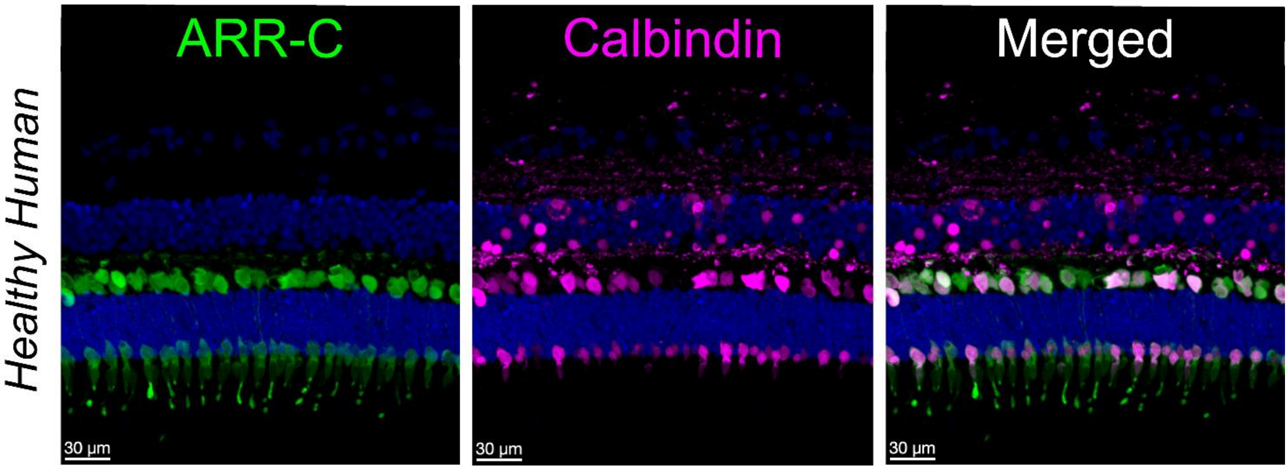

Figure S2: Composite image generated using IBEX demonstrates ARR-C labelling to illustrate cones and calbindin within the inner segment region of a healthy human central retina. Calbindin expression is observed within the inner segments of ARR-C+ cones.

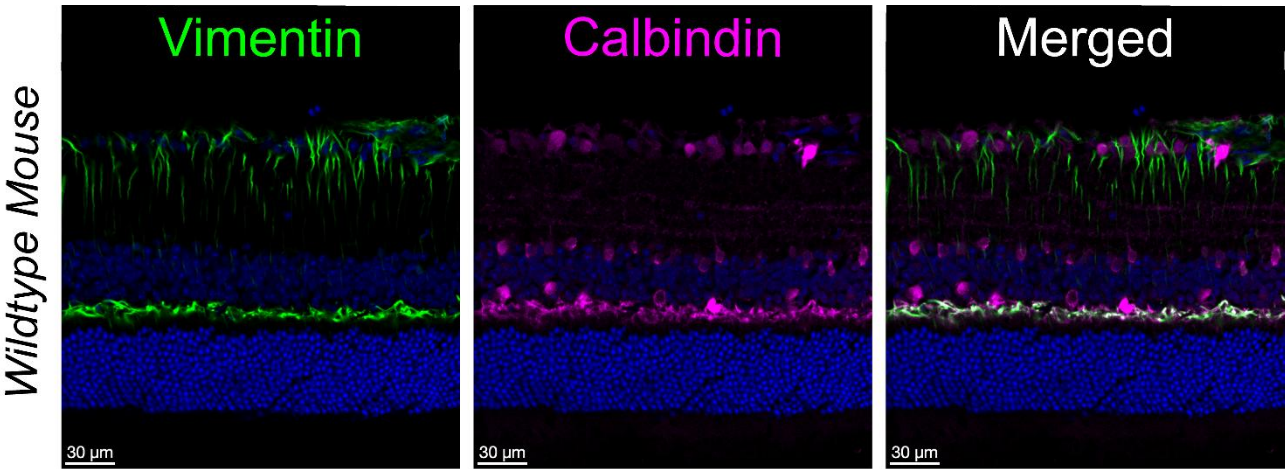

Figure S3: Composite image generated using IBEX shows Vimentin and Calbindin labelling in a wildtype mouse retina. Vimentin expression in mouse retina at the OPL region colocalises with calbindin.

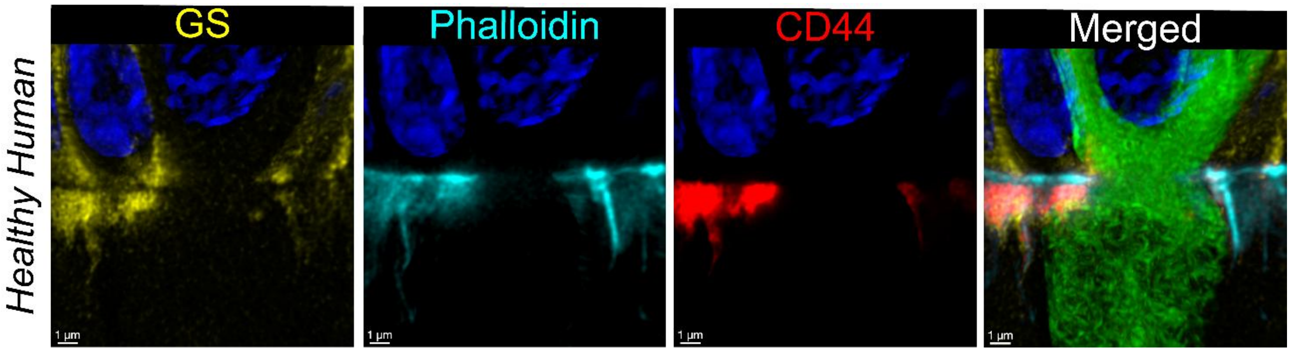

Figure S4: Super-resolution imaging of the GS-Phalloidin-CD44 complex in the human retina. A linear gap can be observed at the endfeet of Müller Glia colocalising with the F-actin-rich adherens belt (Phalloidin)

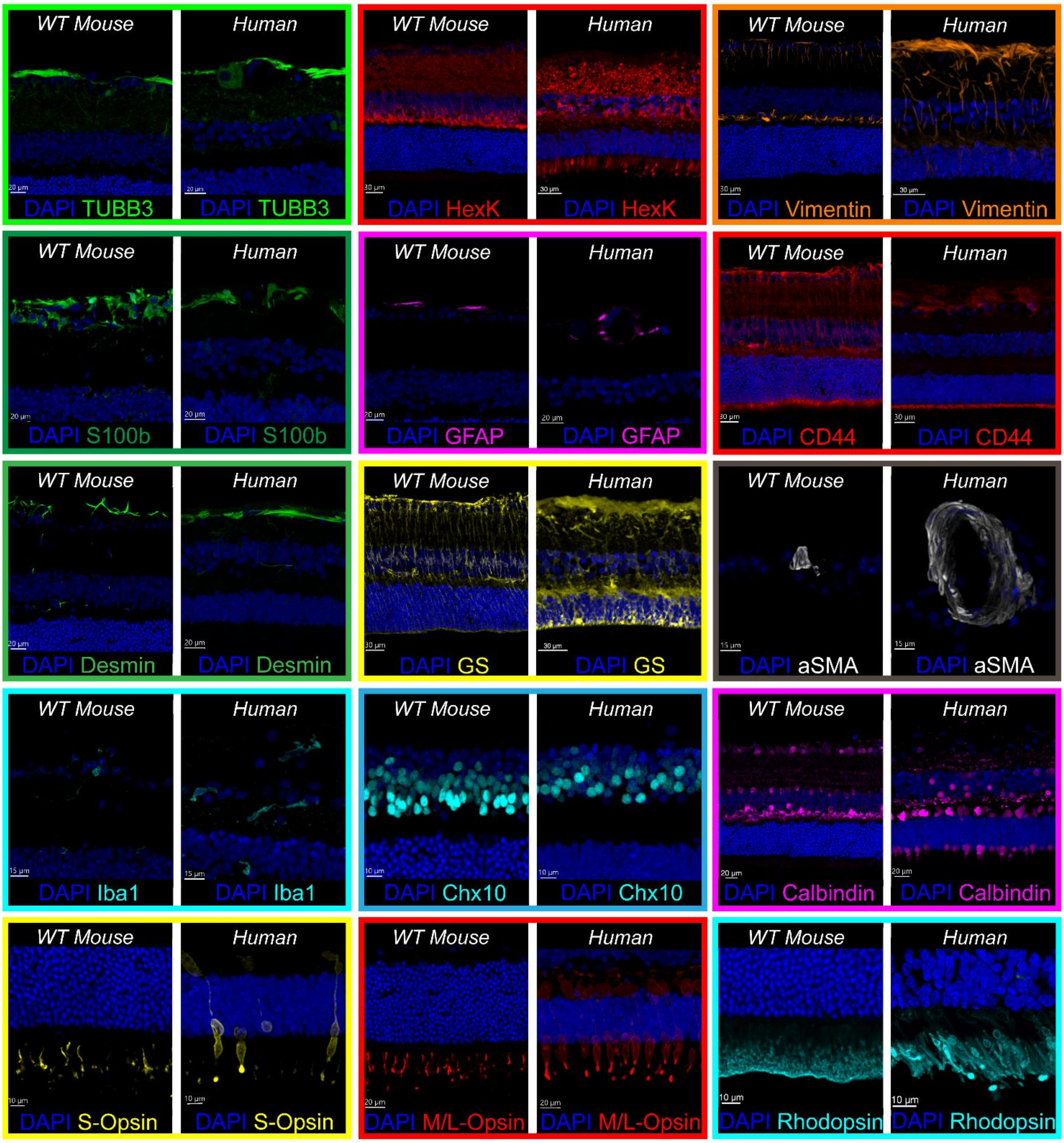

Figure S5: Cross-reactive protein marker comparisons in wildtype mouse and healthy human retinas. All images of the fifteen cross-reactive markers were generated using IBEX.

Table S1 Primary antibodies and lectins used in immunohistochemistry for fixed-frozen human retina

Marker	Clone	Conjugate	Isotype	Vendor	Cat. Number	Dilution	RRID
DAPI	-	UV	N/A	Merck-Sigma	D9542-1MG	1:1000	-
GS	GS-6	AF532	Mouse IgG2a,κ	Merck-Sigma	MAB302	1:100	AB 2110656
Iba1	-	Purified	Goat IgG	Abcam	Ab5076	1:200	AB 2224402
αSMA	IA4	AF532	Mouse IgG2a,κ	ThermoFisher	14-9760-82	1:100	AB 2572996
Desmin	Y66	AF488	Rabbit IgG	Abcam	Ab185033	1:200	AB 2892748
Calbindin	-	AF532	Rabbit Polyclonal	Merck-Sigma	ABN2192	1:50	AB 2935805
PKCa	H-7	AF680	Mouse IgG1,κ	Santa-Cruz	SC-8393	1:100	AB 628142
ChAT	-	Purified	Goat Polyclonal	Merck-Sigma	AB144P	1:100	AB 2079751
β3-Tubulin	TUJ1	AF488	Mouse IgG2a,κ	Biolegend	801203	1:200	AB 2564757
Chx10	E-12	AF546	Mouse IgG2a,κ	Santa-Cruz	SC-365519	1:100	AB 10842442
GFAP	SMI25	AF488	Mouse IgG2b,κ	Biolegend	837508	1:200	AB 2734610
Vimentin	O91D3	AF555	Mouse IgG2a,κ	Biolegend	677802	1:200	AB 2565982
CD44	IM7	i594	Rat IgG2b,κ	AAT Bioquest	356128	1:100	AB 2935685
HSP60	-	Purified	Rabbit Polyclonal	Abcam	ab46798	1:200	AB 881444
Arrestin C	7G6	AF488	Mouse IgG1,κ	Merck-Sigma	MABN2636	1:200	AB 2935804
S-Opsin	-	AF532	Rabbit Polyclonal	Merck-Sigma	AB5407	1:100	AB 177457
STING	E9X7F	AF647	Rabbit IgG	CST	90173	1:100	AB 3675228
S100β	EP1576Y	AF488	Rabbit IgG	Abcam	Ab196442	1:200	AB 2722596
YH2AX	-	AF546	Rabbit Polyclonal	ProteinTech	10856-1-AP	1:200	AB 2114985
M/L-Opsin	-	i594	Rabbit Polyclonal	Merck-Sigma	Ab5405	1:200	AB 177456
COL IV	1042	e660	Mouse IgG2b,κ	ThermoFisher	50-9871-82	1:200	AB 2574404
PNA	-	AF488	N/A	ThermoFisher	L32458	1:200	-
Rhodopsin	1D4	AF546	Mouse IgG1,κ	Santa-Cruz	SC-57432	1:200	AB 785511

Table S2 Primary antibodies and lectins used in immunohistochemistry for fixed-frozen mouse retina

Marker	Clone	Conjugate	Isotype	Vendor	Cat. Number	Dilution	RRID
DAPI	-	UV	N/A	Merck-Sigma	D9542-1MG	1:1000	-
Desmin	Y66	AF488	Rabbit IgG	Abcam	Ab185033	1:200	AB 2892748
S-Opsin	-	AF532	Rabbit Polyclonal	Merck-Sigma	AB5407	1:100	AB 177457
Chx10	E-12	AF546	Mouse IgG2a,κ	Santa-Cruz	SC-365519	1:100	AB 10842442
Hexokinase	C35C4	i594	Rabbit IgG	CST	2024S	1:100	AB 2116996
STING	E9X7F	AF647	Rabbit IgG	CST	90173	1:100	AB 3675228
CD45	30-F11	AF700	Rat IgG2b,κ	Biolegend	103128	1:100	AB 493715
S100β	EP1576Y	AF488	Rabbit IgG	Abcam	Ab196442	1:200	AB 2722596
Calbindin	-	AF532	Rabbit Polyclonal	Merck-Sigma	ABN2192	1:100	AB 2935805
HSP60	-	Purified	Rabbit Polyclonal	Abcam	ab46798	1:200	AB 881444
M/L-Opsin	-	i594	Rabbit Polyclonal	Merck-Sigma	Ab5405	1:200	AB 177456
GFAP	SMI25	AF647	Mouse IgG2b,κ	Biolegend	837511	1:200	AB 2734610
PKCa	H-7	AF680	Mouse IgG1,κ	Santa-Cruz	SC-8393	1:100	AB 628142
GS	GS-6	AF532	Mouse IgG2a,κ	Merck-Sigma	MAB302	1:100	AB 2110656
Iba1	-	Purified	Goat IgG	Abcam	Ab5076	1:200	AB 2224402
CD44	IM7	i594	Rat IgG2b,κ	AAT Bioquest	356128	1:100	AB 2935685
β3-Tubulin	TUJ1	AF488	Mouse IgG2a,κ	Biolegend	801203	1:200	AB 2564757
P53	7F5	Purified	Rabbit IgG	CST	2527S	1:100	AB 10695803
PNA	-	AF488	N/A	ThermoFisher	L32458	1:200	-
αSMA	IA4	AF532	Mouse IgG2a,κ	ThermoFisher	14-9760-82	1:100	AB 2572996
p16	F-12	AF546	Mouse IgG2a,κ	Santa-Cruz	SC-1661	1:200	AB 628067
Isolectin B4	-	AF594	N/A	ThermoFisher	I21413	1:200	-
Calretinin	DAK-Calret1	AF647	Mouse IgG1,κ	Dako Agilent	M724529-2	1:200	AB 2068519
COL IV	-	Purified	Rabbit Polyclonal	Abcam	ab19808	1:100	AB 445160
Rhodopsin	1D4	AF546	Mouse IgG1,κ	Santa-Cruz	SC-57432	1:200	AB 785511
Vimentin	W16220A	AF647	Rat IgG2a,κ	Biolegend	699308	1:200	AB 2888890

Table S3 Secondary Antibodies used for immunohistochemistry

Marker	Clone	Conjugate	Isotype	Vendor	Cat. Number	Dilution	RRID
anti-Rabbit IgG	-	AF488	Donkey IgG	ThermoFisher	A21206	1:1000	AB 2535792
anti-Rabbit IgG	-	AF594	Donkey IgG	ThermoFisher	A32754	1:1000	AB 2762827
anti-Rabbit IgG	-	AF680	Donkey IgG	ThermoFisher	A32802	1:1000	AB 2762836
anti-Rat IgG	-	AF594	Donkey IgG	ThermoFisher	A21209	1:1000	AB 2535795
anti-Goat IgG	-	AF555	Donkey IgG	ThermoFisher	A32816	1:1000	AB 2762839
